# An exploration of the influence of ZnO NPs treatment on germination of radish seeds under salt stress based on the YOLOv8-R lightweight model

**DOI:** 10.1186/s13007-024-01238-8

**Published:** 2024-07-23

**Authors:** Zhiqian Ouyang, Xiuqing Fu, Zhibo Zhong, Ruxiao Bai, Qianzhe Cheng, Ge Gao, Meng Li, Haolun Zhang, Yaben Zhang

**Affiliations:** 1 College of Engineering, Nanjing Agricultural University, Nanjing, 210031 China; 2 Institute of Farmland Water Conservancy and Soil-Fertilizer, Xinjiang Academy of Agricultural and Reclamation Science, Shihezi, 832000 Xinjiang China

**Keywords:** Radish seeds, YOLO model, Germination test, ZnO NPs, Salt stress

## Abstract

**Background:**

Since traditional germination test methods have drawbacks such as slow efficiency, proneness to error, and damage to seeds, a non-destructive testing method is proposed for full-process germination of radish seeds, which improves the monitoring efficiency of seed quality.

**Results:**

Based on YOLOv8n, a lightweight test model YOLOv8-R is proposed, where the number of parameters, the amount of calculation, and size of weights are significantly reduced by replacing the backbone network with PP-LCNet, the neck part with CCFM, the C2f of the neck part with OREPA, the SPPF with FocalModulation, and the Detect of the head part with LADH. The ablation test and comparative test prove the performance of the model. With adoption of germination rate, germination index, and germination potential as the three vitality indicators, the seed germination phenotype collection system and YOLOv8-R model are used to analyze the full time-series sequence effects of different ZnO NPs concentrations on germination of radish seeds under varying degrees of salt stress.

**Conclusions:**

The results show that salt stress inhibits the germination of radish seeds and that the inhibition effect is more obvious with the increased concentration of NaCl solution; in cultivation with deionized water, the germination rate of radish seeds does not change significantly with increased concentration of ZnO NPs, but the germination index and germination potential increase initially and then decline; in cultivation with NaCl solution, the germination rate, germination potential and germination index of radish seeds first increase and then decline with increased concentration of ZnO NPs.

## Background

As the basic means of plant reproduction and dissemination, seeds are an important means of agricultural production, as their quality directly affects the yield and quality of crops [1, 2]. The germination of seeds is an important indicator of seed quality. The monitoring of seed quality can lay the foundation for cultivating high-quality seeds and increasing food production [3, 4].

Radish is a root vegetable which originated from the cruciferous radish family in Europe [5]. It has high edible and medical value with delicious taste [6]. Due to environmental degradation, climate change, etc., the abiotic stress on growth and productivity of plants has been aggravated [7]. Salt stress is a major abiotic stress that interfere with the growth and development of plants. zinc oxide nanoparticles (ZnO NPs) is one of the most widely used engineering nanomaterials [8] with certain biological toxicity [9]. With the advancement and widespread application of nanotechnologies, the release of ZnO NPs into environment has been inevitable, which poses a continuous threat to the ecological safety of plants [10]. Yang et al. [11] found that ZnO NPs with a mass concentration of 2000 mg/L significantly inhibited the elongation of root systems of corn and rice. At the same time, however, ZnO NPs also has many positive effects on growth of plants. Li et al. [12] found that the application of ZnO NPs can reduce the toxicity of Cd to rice seedlings, and promote the increase of seedling weight, total fresh weight, and root-to-crown ratio. However, the effect of ZnO NPs treatment on the salt resistance of radish seeds has rarely been reported. The exploration of the effects of single zinc oxide nanoparticles treatment and single salt stress on the germination of radish seeds, and the effects of zinc oxide nanoparticles treatment on the resistance of radish seeds can provide a test basis for cultivating stress-resistant varieties and a scientific basis for rational use of nanomaterials in agriculture, thereby laying the foundation for sustainable agricultural development.

Traditional germination testing of seeds requires manual counting, which has problems such as slow efficiency, strong subjectivity, and proneness to error. What’s more, some chemical testing methods are destructive to seeds and cause infection, etc. [13], such as staining method [14], conductivity method [15], enzymatic method [16]. At present, there are many tests for the determination of seed vitality. Hampton et al. [14] invented the rapid tetrazole staining method, where the germination and emergence ability of seeds are determined based on the strength of metabolism judged through differences in red characteristics due to dehydrogenation and reduction reaction and the inaccurate judgment caused by seed dormancy is avoided. Borji et al. [17] tested the conductivity of soybean leaching solution and found that there is a very significant negative correlation between conductivity and germination rate; Wang et al. [18] adopted artificial accelerated aging method, it was found that the control of moisture content of wolfberry seeds near (5.70 ± 1)% can maintain high vitality and anti-aging ability of seeds stored at low temperature.

In recent years, non-destructive testing of seed germination has been a trend [4], including include near-infrared spectroscopy [13], infrared thermal imaging [19], electronic nose [20], laser speckle technology [21], etc. He et al. [13] used near-infrared spectroscopy to identify the vitality of 2,400 rice seeds harvested in three different years with a high classification accuracy of 93.67%, thereby developing a fast and cost-effective industrial online seed sorting system; Zhang et al. [20] optimized the electronic nose-based sensor array, and used the sensors to classify 5 kinds of sweet corn seeds with different vitality, where the accuracy of the training set and the verification set reached 98.55% and 96.67%, respectively.

Owing to the rapid development of computers, deep learning-based image processing based has been widely used in detecting seed vitality. Genze et al. [22] proposed a machine learning model based on Faster R-CNN, which achieved a high mean average precision (mAP) on a hold-out test data set of approximately 97.9%, 94.2% and 94.3% for Zea mays, Secale cereale and Pennisetum glaucum respectively. Nehoshtan et al. [23] proposed a universal seed germination prediction tool that uses an artificial neural network algorithm in deep learning to classify the germination ability and usability of seeds by analyzing RGB image data. Toda et al. [24] generated a large number of images through the concept of domain randomization, randomly arranged seed objects on a virtual canvas, and successfully used synthetic data to train the instance segmentation neural network model Mask R-CNN to perform phenotypic analysis of the morphology of various crops. Zhao et al. [25] developed a convolutional neural network (YOLO-r) that can detect the germination status of rice seeds and allows automatic evaluation of the total number of germinations, with an average accuracy of 0.9539. The average test time of a single image was 0.011s, and the average absolute error in prediction of germination rate was within 0.1. Zhang et al. [26] proposed a mask R-CNN model trained with microscopic images of tree peony pollen for fast testing of the pollen germination rate and pollen tube length. The R2 value of the linear regression model of tested pollen germination rate and ground conditions was 0.909, and the R2 value of the average pollen tube length was 0.958. By combining machine vision technology and deep learning, Bai et al. [27] constructed a seed germination discrimination model DB-YOLOv5 based on YOLOv5, which was used for fast testing of germination rate, germination potential, germination index and average germination days of wheat seeds. The accuracy rate of the DB-YOLOv5 model for wheat seed germination discrimination was 98.5%. Jiang et al. [28] proposed a YOLOv8-Peas model whose number of parameters, amount of calculation, and weight file sizes were 1.17 M, 3.2 g, and 2.7 MB, respectively. Compared with YOLOv8, they decreased by 61.2%, 61%, and 56.5%, respectively. PEG6000 was used to simulate different drought environments to analyze the germination of peas of different genotypes, and the pea varieties with the best drought resistance were selected.

In summary, the traditional testing of seed vitality cannot cater agricultural automation due to the disadvantages. Therefore, a future trend is to develop lightweight deep learning network models that allow fast and accurate measurement of the effects of different abiotic stresses on seed germination. In this paper, an improved YOLOv8n-based lightweight YOLOv8-R model is proposed. This model significantly reduces the amount of calculation and improves the testing speed while basically maintaining the accuracy through replacement of backbone network with PP-LCNet, the original C2f of the neck with OREPA, the original SPPF with FocalModulation and the original Detect of the head with LADH and the adoption of cross-scale feature fusion module (CCFM) at the neck. The germination tests of radish seeds were carried out under salt, drought and zinc oxide nanoparticle conditions, with the use of seed germination phenotype collection system to achieve the full time-series sequence tracking of germination rate, germination index and germination potential. Then, based on the acquired seed germination image and YOLOv8-R model, the change law of radish seed germination index under different conditions was analyzed.

## Methods

### Seed germination phenotypic Collection System

**Fig. 1:**
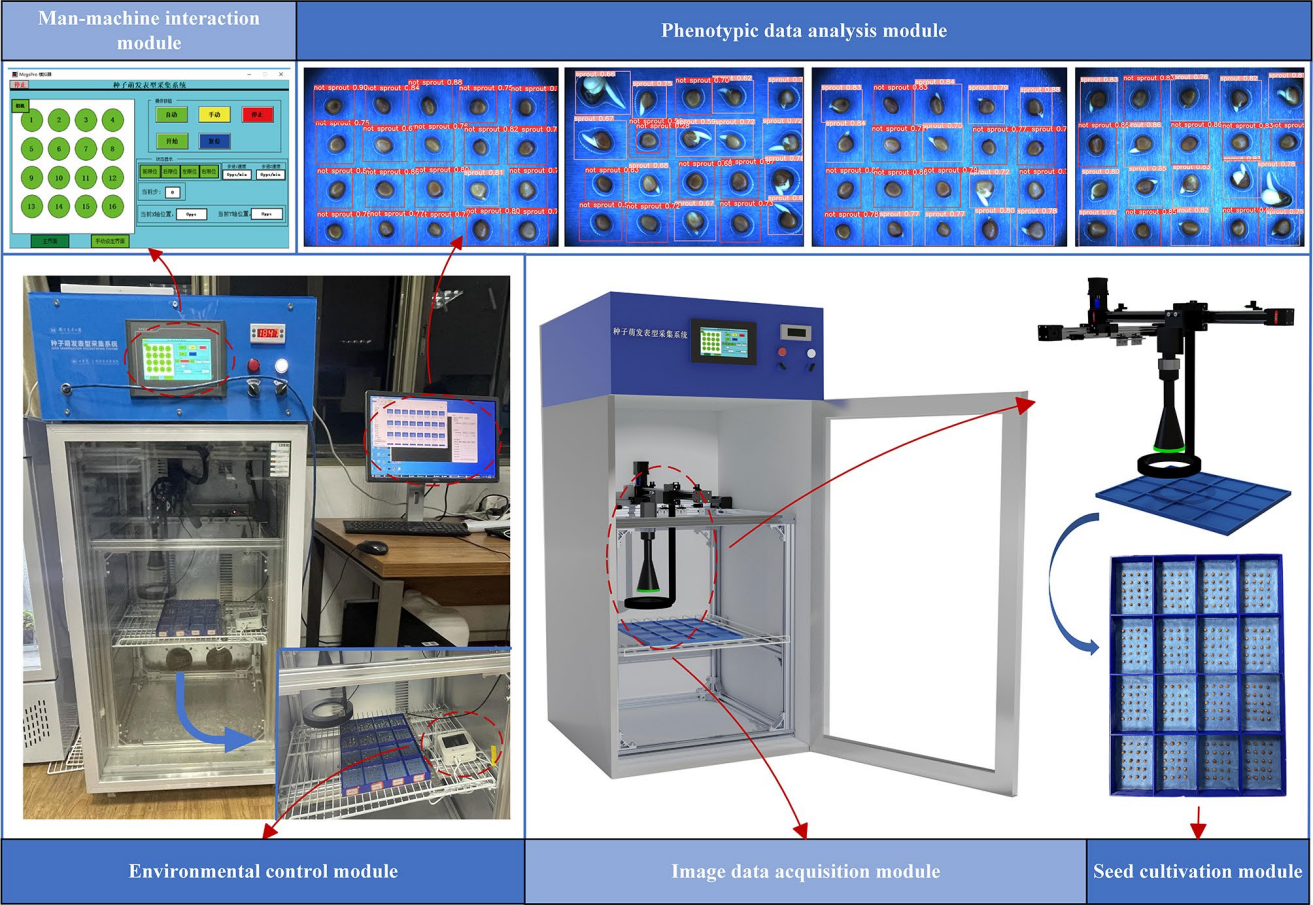
Seed germination phenotypic collection system

As shown in Fig. 1, the experimental seed germination phenotypic acquisition system can be divided into seed cultivation module, environmental control module, image data acquisition module, man-machine interaction module, and phenotypic data analysis module according to functional partitions.

In the seed cultivation module, germination test of radish seeds can be carried out in a 3D-printed 16-cell seed germination petri dish; the environmental control module can realize real-time control of dynamic temperature and humidity of the incubator, where the temperature range is 10 °C to 75 °C, the humidity range is 30–70%. What’s more, the light in the box can also be adjusted by switching; the image data acquisition module consists of an X track and a Y track which are 160 mm above the petri dish, as well as a fixed-focus RGB imaging sensor moving on the tracks. During shooting, the external LED lighting ring is automatically turned on to ensure the clarity of the pictures. The PLC program controls the imaging sensor to collect images of 16 cells in sequence at regular intervals; the human-computer interaction module allows the control of temperature, humidity, shooting interval, and camera movement through the touch screen on the incubator; the phenotypic data analysis module allows the transmission of collected images to the PC for processing through GigE gigabit network high-speed interface.

### Data set construction

**Fig. 2:**
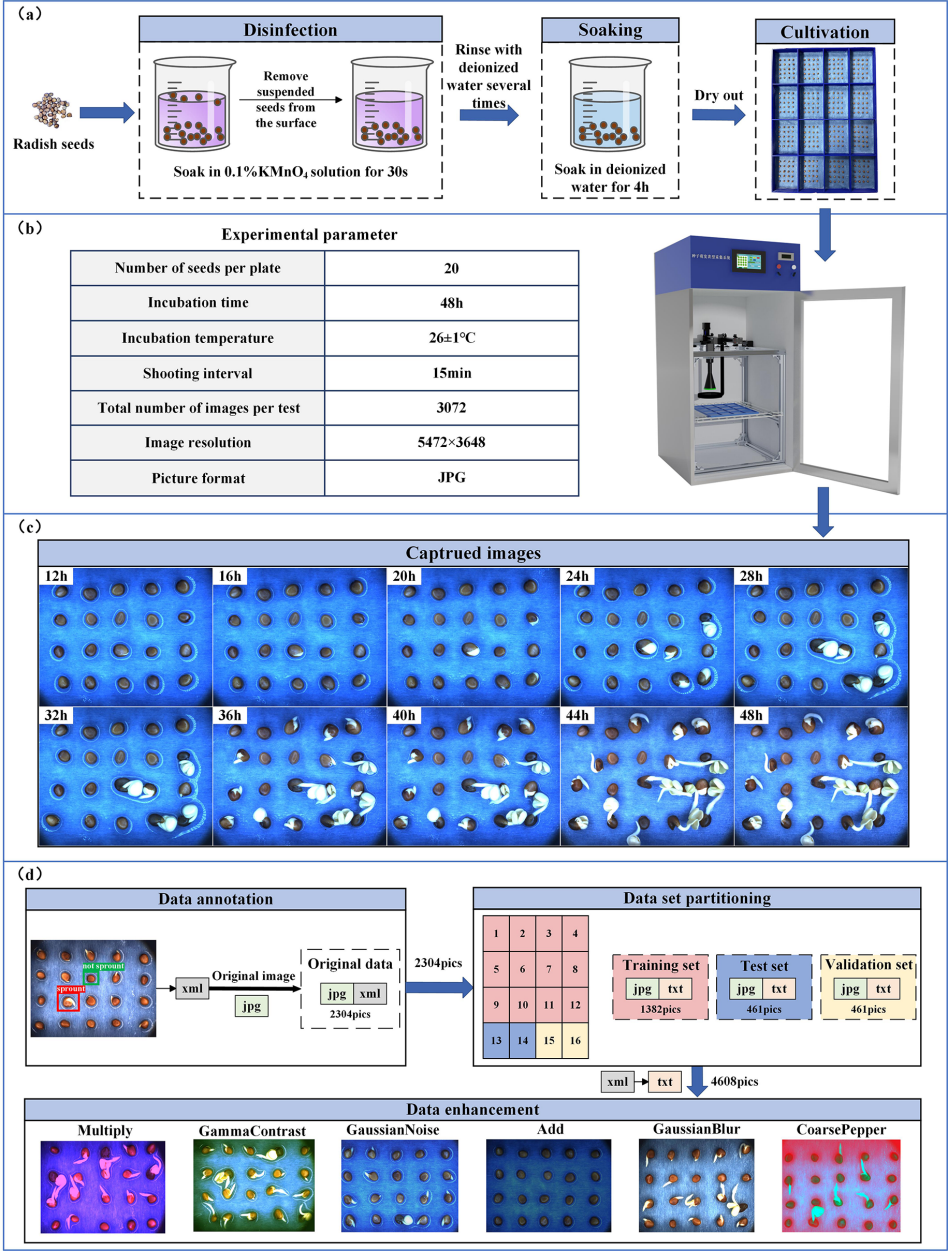
**(a)** Germination test process. **(b)** Test parameters. **(c)** Collected images of radish seed germination. **(d)** Data set construction process

320 full-grain short-leaf early radish seeds without pests or damage were chosen for germination experiments. The germination process is shown in Fig. 2 (a), and the test parameters are shown in Fig. 2(b). The camera collected one image of each of the 16 cells at an interval of 15 min. The experiment lasted for 48 h, and a total of 3072 images were collected. Some of the collected images are shown in Fig. 2(c). It can be seen that the germinated of seeds in the first 12 h was not obvious. Therefore, the 768 images collected in the first 12 h were deleted because they were of little help to the training model, and 2304 original images were left. Next, the data set was build, as shown in Fig. 2 (d).

Germinate was judged by “whitening” of the radish seeds. LabelImg was used to mark the collected pictures as “sprout” or “not sprout”. Then, an xml file containing the image size, the quadrangular coordinates of the label box, and the name of each label box was generated.

The class imbalance occurs when the non-germinating category (majority category) accounts for a large proportion in this dataset, while the germinating category (minority category) has a small sample size. This imbalance may result in a model with good predictive performance for most classes, but poor predictive performance for a few classes, ultimately affecting the performance of the overall model. In unbalanced data sets, random segmentation may lead to uneven distribution of classes in training set, test set and validation set. This means that certain categories may be underestimated in the training set, resulting in poor predictive performance of the model for those categories. For minority samples, random segmentation may lead to a further reduction in the number of minority samples in the training set, and the model may not be able to fully learn the features of the minority class. In this case, the model is more likely to overfit to majority category and ignore minority category. When evaluating the performance of the model, the unbalanced distribution of categories in the test set will lead to the unreliability of the evaluation indexes (such as Precision, Recall, etc.). For unbalanced datasets, when dividing training sets, test sets, and validation sets, stratified sampling can be used to ensure that the distribution of categories in each set is similar to the original data set. Stratified sampling can also avoid the evaluation bias caused by class imbalance and improve the reliability of model evaluation results. Genze et al. [22] adopted the method of petri dish stratification to ensure that seeds of the same petri dish are in the training set, test set or verification set, while ensuring that petri dishes at different time points only appear in one set.

We divide all images of culture dishes 1–12 into training set, all images of culture dishes 13–14 into test set, and all images of culture dishes 15–16 into validation set. The ratio of the number of images in the training set, test set, and validation set is 6:2:2. Since the number of germination categories and non-germination categories in each culture dish at each time point is similar, this division ensures that the category distribution in the training set, test set, and validation set is similar to that of the original dataset.

In order to improve the robustness and generalization of the network model, the collected original images were enhanced to increase the number and diversity of samples. The enhancement methods in the experiment include: Add, Multiply, GaussianBlur, CoarsePepper, GammaContrast, and GaussianNoise. After the enhancement, a total of 4608 images, including the original images, were obtained. The training set, test set, and validation set have 2764, 922, and 922 images, respectively.

### YOLOv8n-based lightweight structure design (YOLOv8-R)

**Fig. 3:**
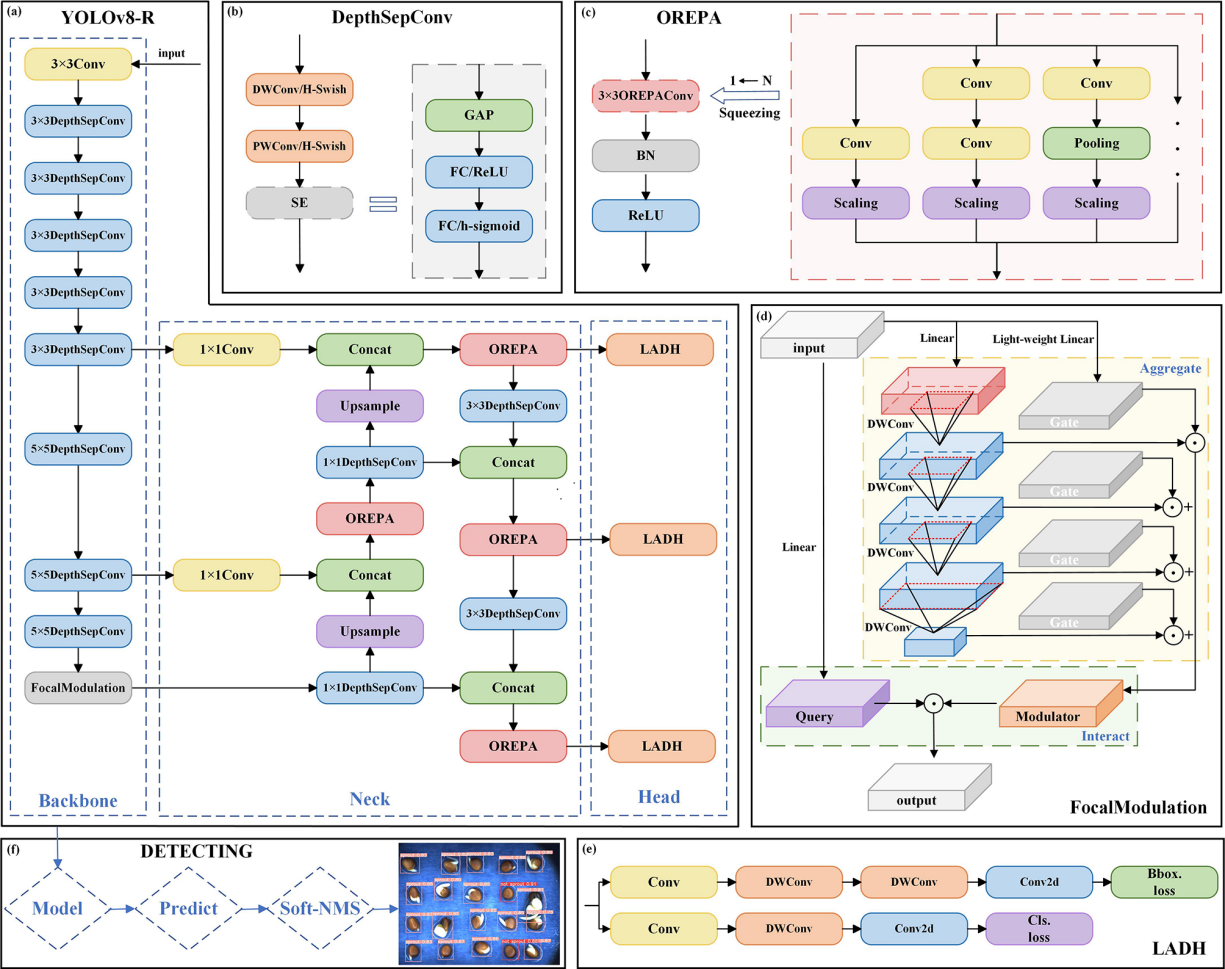
**(a)** YOLOv8-R network structure. **(b)** DepthSepConv network structure. **(c)** OREPA network structure and block extrusion process. **(d)** FocalModulation network structure and aggregation process. **(e)** Lightweight asymmetric detection head network structure. **(f)** Post-processing step framework

The YOLO series has achieved great success in the field of computer vision. As the latest target test model of YOLO series, YOLOv8 exhibits excellent robustness and strong learning ability, being the fastest and most accurate target testing model in this series [29]. In the actual agricultural production, due to the problems such as limited computing power of equipment, it is necessary to reduce the number of parameters and computational complexity of the model while maintaining high precision, so that the model can be deployed on embedded or mobile platforms. Based on YOLOv8n which balances detection accuracy and model complexity, a YOLOv8-R model for testing the germination of radish seeds is proposed. Its network structure is shown in Fig. 3(a), and the specific improvements are as follows:

(1) Replacement of backbone network (P-YOLOv8) with PP-LCNet: The backbone network is a key component for extracting data features. The original backbone network structure of YOLOv8n is complex, which resulted in excessive parameters and increased calculations. In PP-LCNet [30], DepthSepConv [31] is used instead of ordinary convolution as the basic module. Since there is no skip connection, this module will not be a significant impact on the inference speed of the model. The network structure of DepthSepConv is shown in Fig. 3(b). It is a fusion of deep convolution (DWConv) and point convolution (PWConv). The use of H-Swish [32] in activation functions of PWConv and DWConv can avoid a large number of exponential operations. The activation functions used in the SE [33] layer are ReLU and h-sigmoid. The image input trunk passes through the 3 × 3Conv module, the 3 × 3DepthSepConv module and the 5 × 5DepthSepConv module for feature extraction. After testing, it was found that replacement of the convolution module of the neck with DepthSepConv can reduce the number of parameters by 0.16 M and the amount of calculation by 0.2G.

(2) Adoption of cross-scale feature fusion module CCFM (PC-YOLOv8) in the neck: CCFM [34] is proposed in the RT-DETR model, and jointly constitutes the encoder of the RT-DETR model with attention-based intra-scale feature interaction (AIFI). The main principle of CCFM is to enhance the model’s adaptability to scale changes and the ability to detect small-scale objects through the fusion of the features of different scales. The effective integration of detailed features and contextual information improves the overall performance of the model. CCFM can address the computational redundancy in the encoder to achieve lightweight RT-DETR model. After reproduction of it in YOLOv8n, it was found in the tests that the number of parameters and amount of calculation of YOLOv8n are also significantly reduced.

(3) Replacement of C2f (PCO-YOLOv8) of the neck with OREPA: OREPA [35] reduces the cost and complexity of model training through online convolution re-parameterization, which is divided into two stages: block linearization and block extrusion. Block linearization is to remove the nonlinear blocks from the prototype re-parameterization blocks, leaving only the convolution layer and the batch normalization (BN) layer, and a scaling layer is added to optimize the performance. Block extrusion is to merge a series of convolutional layers, pooling layers, scaling layers, and frequency prior layer into a single convolution (OREPAConv). No matter how complex the model is during training, they will be eventually be compressed into a single 3 × 3OREPAConv, thereby reducing resource loss. Through the dynamic adjustment of the weight of the convolutional layer, OREPA greatly reduces the complexity of the model while ensuring the accuracy. Its structure and the process of block extrusion are shown in Fig. 3(c).

(4) Replacement of SPPF (PCOF-YOLOv8) with FocalModulation: FocalModulation [36] is a feature enhancement method that adopts an attention mechanism to focus on key areas of an image, thereby improving the model’s ability to recognize these areas. In FocalModulation, the input is first processed through the linear layer. Then, the information is aggregated selectively through Hierarchical contexalization [37] and Gated aggregation [38]. Finally, the output is generated through interaction between the modulator and query. Hierarchical contexalization is to extract contextual features of different granularity levels through stacking of DWConv layers, while gated aggregation is to aggregate contextual features into a modulator. FocalModulation technology is used to replace the original spatial pyramid pooling-fast (SPPF), which improves the accuracy of the network model without changing the number of parameters and amount of calculation. It is a spatial pyramid pooling with higher accuracy. The structure of FocalModulation and the aggregation process are shown in Fig. 3(d).

(5) Replacement of Detect (YOLOv8-R) with LADH [39]. The traditional YOLO detector usually adopts a symmetric multi-level compression structure, that is, the compression ratio of each detection head is the same. The Lightweight Asymmetric Dual-Head (LADH) structure adopts asymmetric multi-level compression, that is, different compression ratios are used for different categories of detection heads, which can better adapt to the feature representation and target size distribution of different categories and improve the generalization ability of the detector. LADH contains Asymmetric Head and Dual-Head. Asymmetric Head is responsible for asymmetric compression of features of different categories; Dual-Head combines the outputs of two Asymmetric Heads and generates the final object detection results. In LDAH, the traditional 3 × 3Conv is replaced by 3 × 3DWConv, which significantly reduces the number of parameters. The structure of LADH is shown in Fig. 3(e).

(6) Post-processing step. The YOLOv8-R model may have false positives and duplicate detections. To solve these problems, post-processing operations are usually performed. Non-Maximum Suppression (NMS) is a standard technique for post-processing of target detection. Soft-NMS [40] is a variant of NMS that alleviates the problem of duplicate detection by reducing the confidence scores of highly overlapping boxes instead of directly deleting them. This can retain more detection boxes while reducing the impact of duplicate detection. Through continuous experiments and adjustments, we set the threshold at 0.01 and the sigma value at 0.5. The training of the YOLOv8-R model is combined with the use of soft-NMS post-processing technology to optimize the results of target detection and positioning in the image. The framework of post-processing step is shown in Fig. 3(f).

### Model training parameters and evaluation indicators

In this experiment, Windows 11 operating system was adopted. The computer was equipped with Intel (R) Xeon(R) Gold 6248R@3.00 GHz processor, 30GB memory, NVIDIA GeForce RTX3090 graphics card and 24GB video memory. The development language was Python3.8. The deep learning model framework was Pytorch2.0.0, and the CUDA version was 11.7. The settings of model training parameters are shown in Table 1.

**Table 1:** Settings of model parameters

Parameter	Value
Epoch	100
Batch Size	16
Image Size	640 × 640
Optimizer	Adam
Learning Rate	10^− 4^
Weight Decay	10^− 4^
Momentum	0.937
Workers	8

In order to comprehensively evaluate the effectiveness of the model in testing the germination of radish seeds and its satisfaction of the lightweight requirements on low cost and high efficiency, the precision rate (P), recall rate (R), and mean average precision (mAP) were used as indicators to evaluate the accuracy of the model; the number of parameters (Params), and floating-point Operations Per second (FLOPs), and weight size (Weight Size) were used as indicators to evaluate the complexity of the model.

The precision rate is the proportion of the correct prediction in all the results predicted by the model. The recall rate is the proportion of the correct prediction in all positive samples. The average precision (AP) is the area under the PR curve. AP is calculated for each category, and the average AP of all categories is taken as mAP [41]. mAP50 represents the average precision when the Intersection over Union (IoU) threshold is 0.5. The parameter quantity is the number of all parameters of the model; the amount of calculation is the total number of floating-point calculation of the model; the weight file contains the weight and bias in each layer of the model and other parameters. All three indicators are used to reflect the degree of lightness of the model. The specific calculation formula is shown below.

1 $$\:P = \frac{{TP}}{{TP + FP}}$$

2 $$\:R=\frac{TP}{TP+FN}$$

3 $$\:AP = \int_0^1 {P \cdot \:RdR}$$

4 $$\:mAP = \frac{1}{C}\sum\nolimits_{i = 1}^C {A{P_i}}$$

5 $$\:IoU=\frac{Intersection\:Area}{Union\:Area}$$

6 $$\:Params={C}_{\text{in\:}}\times\:{K}^{2}\times\:{C}_{\text{out\:\:}}$$

7 $$\:FLOPs=2\times\:H\times\:W\left({C}_{in}{K}^{2}+1\right){C}_{\text{out\:}}$$

Where, true positives (TP) means the number of germinated and non-germinated seeds correctly detected by the model; false positives (FP) means false identification of non-seed areas as the number of seeds marked “sprouted” or “not sprouted” by the model; false negatives (FN) means the actual number of germinated or non-germinated seeds that are not detected by the model; The AP_i_ means the AP value with category index value i; C means the number of categories; The Intersection Area is the overlapping area of the predicted box and the true box, and the Union Area is the total area of the two boxes minus the Intersection Area. $\:{\text{C}}_{\text{in\:}}$and $\:{\text{C}}_{\text{out\:\:}}$mean the number of input and output channels; H and W mean the spatial size of the output feature map; and K means the convolution kernel size.

### Ablation tests

In order to verify the feasibility of the proposed improved model YOLOv8-R in terms of performance, and reflect the impact of different improved modules on its detection performance, ablation tests were carried out based on YOLOv8n. In each training, all parameters were consistent except the improvement of modules. The ablation test results are shown in Table 2.

**Table 2:** Ablation test results

Model	*P*/%	*R*/%	mAP50/%	Params/M	FLOPs/G	Weight Size/MB
YOLOv8n	99.2	99.1	99.2	3.01	8.1	6.2
P-YOLOv8	96.5	98.0	98.8	1.69	5.0	3.4
PC-YOLOv8	96.7	98.1	98.7	0.75	3.6	1.7
PCO-YOLOv8	96.3	97.6	98.7	0.94	3.2	2.1
PCOF-YOLOv8	98.0	98.8	99.1	0.93	3.2	2.0
YOLOv8-R	98.3	98.5	99.2	0.85	3.0	1.8

As shown in Table 2, after the backbone network is replaced with PP-LCNet, the presence of DepthSepConv reduced a large number of operations. The number of parameters, amount of calculation, and weight file size of P-YOLOv8 were reduced by 43.9%, 38.3% and 45.2%, respectively, but the detection accuracy was sacrificed. After adoption of CCFM in the neck part, the computational redundancy was effectively eliminated, which further reduced the number of parameters, amount of calculation, and weight file size of PC-YOLOv8 by 55.6%, 28% and 50%, respectively while basically maintaining the precision. After the C2f of the neck part was replaced with OREPA, the complex structure can be compressed into a single convolution. The number of parameters and weight file size of PCO-YOLOv8 slightly increased, but the amount of calculation was reduced by 11.1% without the detection of precision. After FocalModulation was replaced with SPPF, the model complexity of PCOF-YOLOv8 remained basically the same, but the precision was improved. Finally, after replacing Detect with LADH, the model complexity is reduced again, and the accuracy is slightly improved. In summary, YOLOv8-R significantly reduces the complexity of the model, with excellent performance in terms of lightweight. At the same time, YOLOv8-R maintains the high-precision performance of the basic model YOLOv8n, which reflects its feasibility in testing the germination of radish seeds and its potential to be deployed on embedded devices.

### Comparative test

In order to further verify the superiority and effectiveness of YOLOv8-R, it is compared with the currently widely used target detection models such as RT-DETR-x, Faster R-CNN, Mask R-CNN, YOLOv3, YOLOv3-tiny, YOLOv5s, YOLOv6-v3.0, YOLOv7, YOLOv7x, YOLOv7-tiny, and YOLOv8s. All parameters were consistent during the experiment, and the results are shown in Table 3.

**Table 3:** Comparative test results

Model	*P*/%	*R*/%	mAP50/%	Params/M	FLOPs/G	Weight Size/MB
RT-DETR-x	94.1	94.6	94.9	65.47	222.5	129.1
Faster R-CNN	92.9	96.6	92.4	41.20	203.7	165.2
Mask R-CNN	94.5	97.3	96.1	44.31	260.2	170.0
YOLOv3	96.5	98.1	98.4	103.69	283.0	198.1
YOLOv3-tiny	96.7	97.9	98.5	12.13	18.9	23.2
YOLOv5s	97.9	98.8	98.4	9.11	23.8	17.7
YOLOv6-v3.0	96.3	97.1	98.5	4.23	11.8	8.3
YOLOv7	98.2	98.4	98.8	37.20	105.1	74.8
YOLOv7x	98.1	98.3	98.7	70.03	188.9	142.1
YOLOv7-tiny	87.4	96.7	97.9	6.02	13.2	12.3
YOLOv8s	99.3	99.5	99.1	11.13	28.7	21.5
YOLOv8-R	98.3	98.5	99.2	0.85	3.0	1.8

First, combined with Tables 2 and 3, YOLOv8 has made many improvements in the model architecture compared to previous versions of the YOLO series, which has improved its detection accuracy; the number of parameters and the amount of calculation have also been further reduced, which enables it to run efficiently in resource-constrained environments; YOLOv8n is the smallest version in the YOLOv8 series and is designed for extremely low-resource environments. Faster R-CNN and Mask R-CNN models are usually large, and deployment on embedded devices and mobile devices may require more optimization and resources. Mask R-CNN has advantages in instance segmentation tasks, but is slower in pure detection tasks. As a Transformer-based target detector, RT-DETR-x has a complex model training process and high computing resource requirements, which is not as efficient as the lightweight design of YOLOv8. In summary, it is optimal to choose YOLOv8n as the improved basic model.

**Fig. 4:**
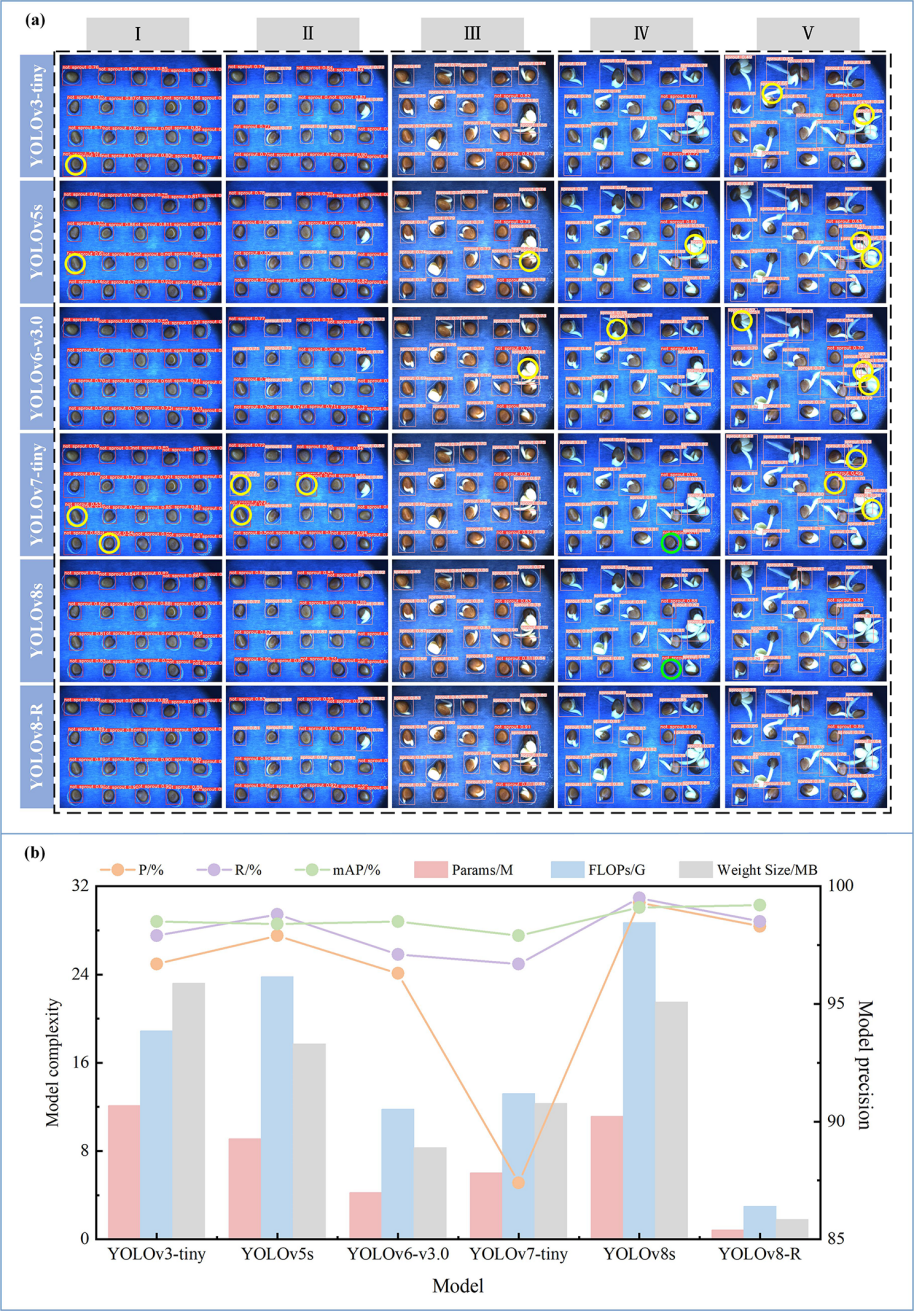
**(a)** The detection results of each lightweight model at different stages of germination. **(b)** Comparison of the performance indicators of each lightweight model

It can be seen from Table 3 that, RT-DETR-x, Faster R-CNN and Mask R-CNN has no advantages in terms of both precision and complexity; YOLOv3, YOLOv3-tiny, YOLOv5s, and YOLOv6-v3.0 exhibited high precision, but YOLOv3 involves a huge amount of calculation and parameters; compared with YOLOv7 and YOLOv7x, YOLOv7-Tiny is lighter, but partial detection precision is sacrificed; compared with the previous YOLO version, YOLOv8s has fewer parameters but better detection performance; the mAP50 of YOLOv8-R is second only to YOLOv8s, and the model complexity is much lower than other models. In order to more intuitively demonstrate the advantages of YOLOv8-R, 6 lightweight models were further compared, including YOLOv3-tiny, YOLOv5s, YOLOv6-v3.0, YOLOv7-tiny, YOLOv8s, and YOLOv8-R. Figure 4 (a) is the detection result of the six models at the five moments of radish seed germination. Where, yellow means repeated detection and green means erroneous detection. At time I, the seeds had not yet germinated, and there was repeated detection of YOLOv3-tiny, YOLOv5s, and YOLOv7-tiny; at time II, the seeds began to germinate, and there was repeated detection of YOLOv7-tiny; at time III, a small part of the seeds were germinated, and there was repeated detection of YOLOv5s, YOLOv7-tiny, and YOLOv8-R; At time IV, most of the seeds were germinated, and there was repeated detection of YOLOv5s and YOLOv6-v3.0. There was erroneous detection of YOLOv7-tiny and YOLOv8s; At time V, all seeds were basically germinated, and repeated detection is found except YOLOv8s. Figure 4(b) more intuitively shows the performance comparison of the above six models. In summary, YOLOv8-R well balance the precision and complexity, and has high overall performance and use value.

## Results and discussion

### Experimental design

**Fig. 5:**
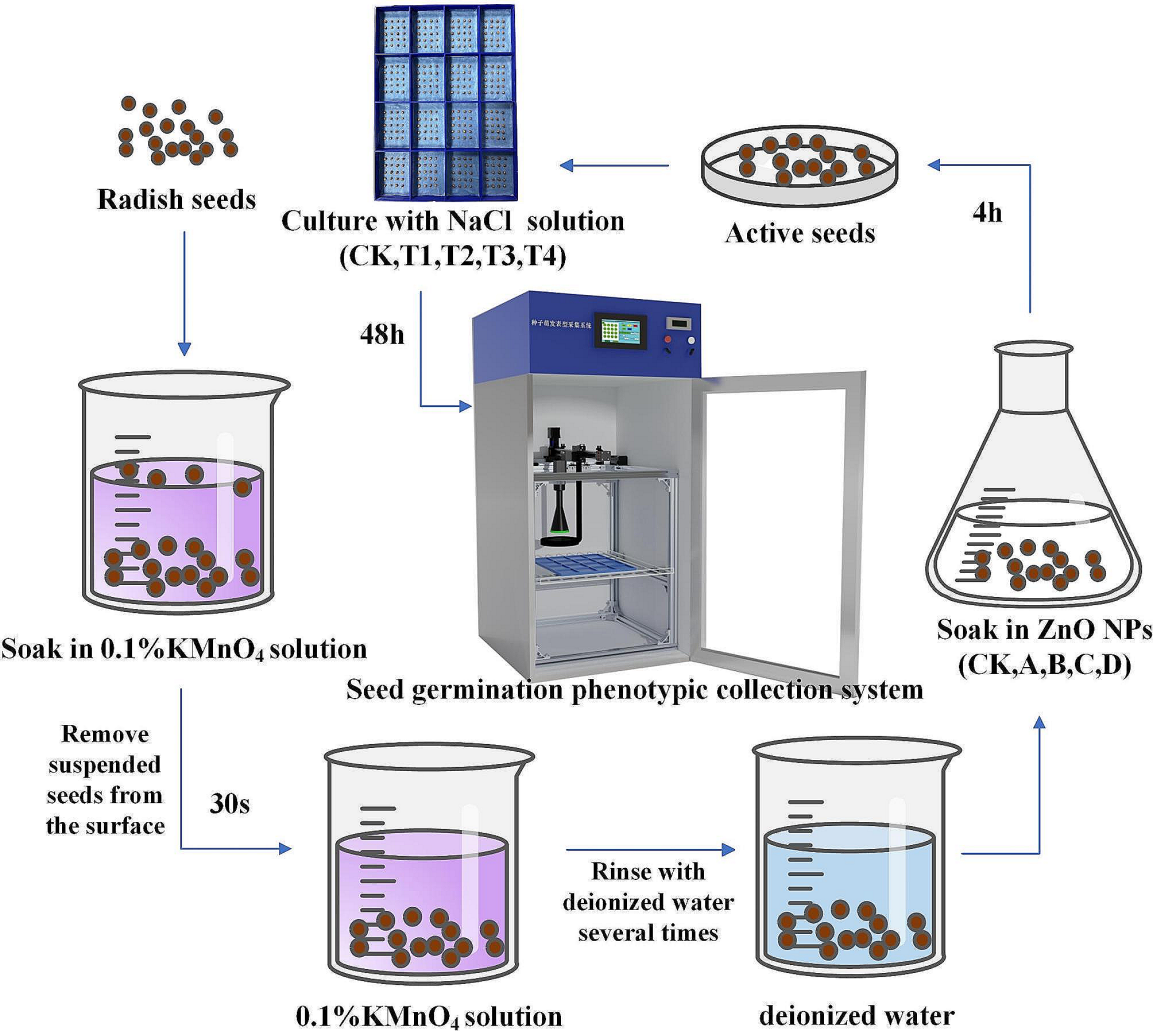
The detection and experimental process of ZnO NPs treatment on the germination of radish seeds under salt stress

Based on the seed germination collection system and the improved target detection model YOLOv8-R, the 48 h full time-series sequence change law of the dual variables of zinc oxide nanoparticles treatment and salt stress on the germination of radish seeds was analyzed. The experimental design is shown in Table 4. The NaCl solution was used to simulate salt stress, and a 2-factor randomized block design was adopted. There were a total of 25 treatments, each of which was done in 4 cells at a time, with a repetition of 3 times. The experimental process is shown in Fig. 5, where the particle size of ZnO NPs was 20 ~ 30 nm.

**Table 4:** Experimental design

Concentration of NaCl/mmol·L^-1^	Concentration of ZnO NPs/mg·L^-1^				
	0(CK)	200(A)	400(B)	600(C)	800(D)
0(CK)	CK	A	B	C	D
30(T1)	T1	AT1	BT1	CT1	DT1
60(T2)	T2	AT2	BT2	CT2	DT2
90(T3)	T3	AT3	BT3	CT3	DT3
120(T4)	T4	AT4	BT4	CT4	DT4

### Evaluation indicators of germination

The germination rate, germination potential, and germination index were used as the evaluation indicators of the germination of radish seeds. The germination potential reflects the germination speed and neatness of seeds, and the germination index can also reflect the germination speed of seeds. The specific calculation formula is as follows:

8 $$\:Germination\:rate=\frac{{N}_{t}}{N}\times\:100\%$$

9 $$\:Germination\:energy=\frac{{N}_{32}}{N}\times\:100\%$$

10 $$\:Germination\:index = \sum {\frac{{{G_t}}}{{{D_t}}}}$$

Where, N_t_ means the number of seeds germinated after t hours; N means the number of tested seeds; N_32_ means the number of seeds germinated within 32 h before the peak of germination; G_t_ is number of seeds germinated after t hours of culture; D_t_ is the corresponding during of culture.

### Effects of different concentrations of ZnO NPs on the germination of radish seeds under salt stress

Salt stress is a key factor that affects growth and yield of crops. Saline soil can cause physiological and metabolic disorders of plants and affect seed germination [42]. At present, the widespread application of nanotechnologies in materials, energy, medicine, and other fields has brough fundamental changes to many aspects of modern society, and its rapid development in biotechnology and agriculture has also been witnessed [43]. However, the influence of ZnO NPs on the germination of radish seeds under salt stress is not yet clear. According to Table 4, the germination rate, germination potential, and germination index of radish seeds treated with ZnO NPs of NaCl concentrations of 0, 30mmol·L^-1^, 60mmol·L^-1^, 90mmol·L^-1^, and 120mmol·L^-1^ were tested and compared to explore the effect of ZnO NPs treatment on the germination of radish seeds under salt stress, thereby providing a test basis for cultivating salt-tolerant varieties.

**Fig. 6:**
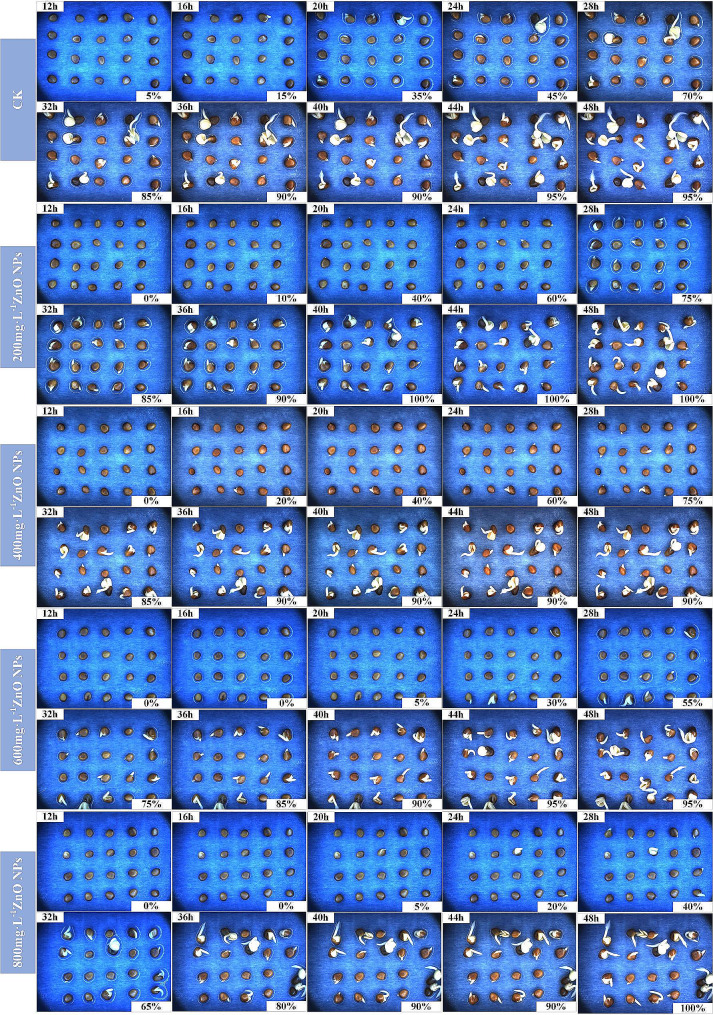
Germination images of radish seeds treated with different concentrations of ZnO NPs

**Fig. 7:**
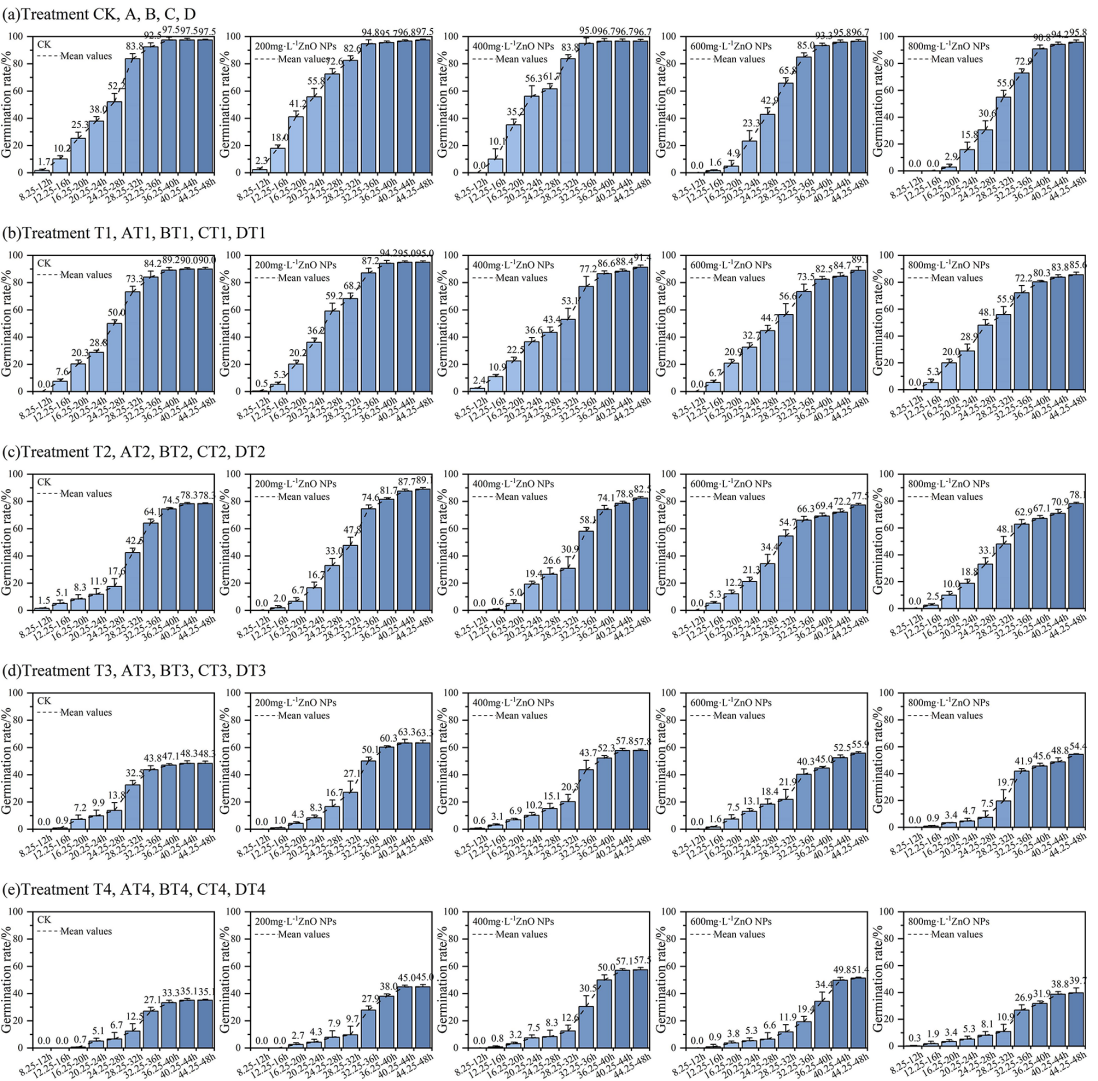
**(a)** CK, A, B, C, D treatment of germination rate. **(b)** T1, AT1, BT1, CT1, DT1 treatment of germination rate. **(c)** T2, AT2, BT2, CT2, DT2 treatment of germination rate. **(d)** T3, AT3, BT3, CT3, DT3 treatment of germination rate. **(e)** T4, AT4, BT4, CT4, DT4 treatment of germination rate

**Fig. 8:**
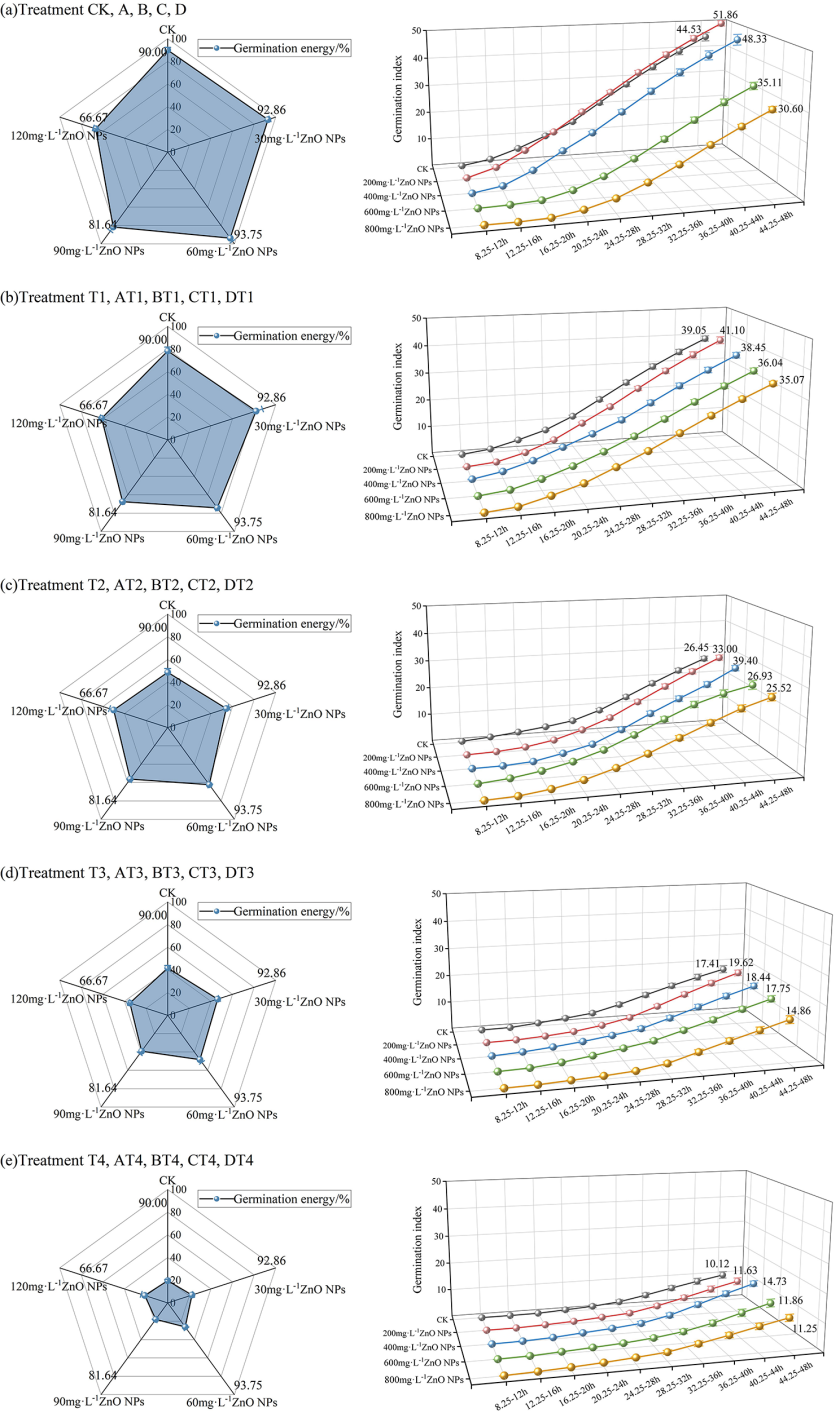
**(a)** CK, A, B, C, D treatment of germination energy and germination index. **(b)** T1, AT1, BT1, CT1, DT1 treatment of germination energy and germination index. **(c)** T2, AT2, BT2, CT2, DT2 treatment of germination energy and germination index. **(d)** T3, AT3, BT3, CT3, DT3 treatment of germination energy and germination index. **(e)** T4, AT4, BT4, CT4, DT4 germination energy and germination index

(1) The effects of treatment with different concentrations of ZnO NPs on the germination of radish seeds. The germination test of radish seeds treated under CK, A, B, C, and D were carried out, as shown in Table 4, and the germination images within 48 h were obtained, as shown in Fig. 6 which shows the continuous germination process of radish seeds and demonstrates the advantages of continuous collection by the phenotype acquisition platform; the YOLOV8-R model was used to detect the germination images, thereby obtaining the germination rate, germination, potential and germination index of radish seeds treated with different concentrations of ZnO NPs, as shown in Figs. 7 and 8.

Figure 7 (a) shows the change in germination rate of radish seeds soaked in different concentrations of ZnO NPs with the advancement of time. Taking the data of Dt = 28.25–32 h as an example, the Mean Value line segment represents the mean germination rate of the three groups of repeated tests during 28.25–32 h. As can be seen from Fig. 7 (a), with the advancement of germination Dt, the germination rate of radish seeds soaked in different concentrations of ZnO NPs showed an increasing trend. With the increase of ZnO NPs concentration, the germination rate of all radish seeds were above 95% without significant difference, but the initial germination time showed a trend of delay and postponement. Within the germination Dt = 44.25–48 h, the germination rates of radish seeds soaked in deionized water (CK), 200mg·L^-1^, 400mg·L^-^1, 600mg·L^-1^, and 800mg·L^-1^ZnO NPs in the control group were 97.50%, 97.50%, 96.67%, 96.67% and 95.83%, respectively. It can be seen that with the increase of ZnO NPs concentration, there is on significant effect on the germination rate of radish seeds.

Figure 8 (a) show the changes in germination index and germination potential of radish seeds soaked in different concentrations of ZnO NPs with the advancement of time. The germination potential of radish seeds soaked in CK, 200mg·L^-1^, 400mg·L^-1^, 600mg·L^-1^, 800mg·L^-1^ZnO NPS was 90.00%, 92.86%, 93.75%, 81.64% and 66.67%, respectively. Within the germination Dt = 44.25–48 h, the germination index of the seeds treated with each concentration was 44.53, 51.86, 48.33, 35.11 and 30.60, respectively. It can be seen that with the increase of ZnO NPs concentration, the germination potential and germination index showed an upward and then downward trend. Where, the germination potential and germination index of seeds treated with ZnO NPs of a concentration of 200mg·L^-1^ and 400mg·L^-1^ were higher than that of the control group; the germination potential and germination index of seeds treated with ZnO NPs of a concentration of 600mg·L^-1^ and 800mg·L^-1^ declined to different degrees compared to those of CK, and the higher the concentration, the more obvious the inhibitory effect. Therefore, the treatment with low-concentration ZnO NPs in breeding radish seeds in deionized water can improve the speed and potential of germination to a certain extent, while high-concentration ZnO NPs will inhibit the speed and potential of germination of seeds.

(2) The effects of different concentrations of ZnO NPs on the germination of radish seeds in 30mmol·L-1NaCl solution. The germination test of radish seeds treated with T1, AT1, BT1, CT1, and DT1 were carried out, as shown in Table 4.

Figure 7 (b) shows the change in germination rate of radish seeds with different concentrations of ZnO NPs soaked in 30mmol·L-1NaCl solution with the advancement time (the same calculation method in (1) is adopted). As can be seen from Fig. 7 (b), with the advancement of germination Dt, the germination rate of radish seeds treated with different concentrations of ZnO NPs in 30mmol·L^-1^NaCl solution showed an increasing trend. The initial germination time of radish seeds soaked in deionized water (CK) in the control group occurred in the first 12.25–16 h, and the initial germination of radish seeds soaked in 400 mg·L-1ZnO NPs occurred in the first 8.25–12 h. The initial germination time showed an earlier trend compared with that of the control group. Within the germination Dt = 44.25–48 h, the germination rates of radish seeds soaked in CK, 200mg·L^-1^, 400 mg·L^-1^, 600mg·L^-1^, and 800mg·L^-1^ZnO NPs were 90.00%, 95.00%, 91.35%, 89.00% and 85.63%, respectively. It can be seen that with the increase of ZnO NPs concentration, the germination rate of radish seeds showed an upward and then downward trend, where the germination rate was the highest when the concentration was 200mg·L^-1^ZnO NPs.

Figure 8 (b) show the changes in germination index and germination potential of radish seeds treated with different concentrations of ZnO NPs in 30mmol·L^-1^NaCl solution with the advancement of time. The germination potential of seeds soaked in CK, 200 mg·L-1, 400mg·L^-1^, 600mg·L^-1^, 800mg·L^-1^ZnO NPS was 78.33%, 81.83%, 74.25%, 67.85% and 61.17%, respectively. Within the germination Dt = 44.25–48 h, the germination index of the seeds treated with each concentration was 39.05, 41.10, 38.45, 36.04 and 35.07, respectively. It can be seen that with the increase of ZnO NPs concentration, both the germination potential and germination index show an upward and then downward trend. Where, the germination potential and germination index of the seeds were the highest when they were soaked in 200 mg·L^-1^ZnO NPs; the germination potential and germination index of the seeds soaked in 400mg·L^-1^, 600mg·L^-1^, and 800mg·L^-1^ZnO NPs were lower at varying degrees compared with those in CK, and the higher the concentration, the more obvious the inhibition effect. Therefore, the treatment of radish seeds with low-concentration ZnO NPs in 30mmol·L^-1^NaCl solution can improve the speed and potential of germination, while high-concentration ZnO NPs will inhibit the speed and potential of germination.

(3) The effects of different concentrations of ZnO NPs on the germination of radish seeds in 60mmol·L^-1^NaCl solution. The germination test of radish seeds treated with T2, AT2, BT2, CT2, and DT2 were carried out, as shown in Table 4.

Figure 7 (c) shows the change in germination rate of radish seeds with different concentrations of ZnO NPs soaked in 60mmol·L^-1^NaCl solution with the advancement time (the same calculation method in (1) is adopted). As can be seen from Fig. 7 (c), with the advancement of germination Dt, the germination rate of radish seeds treated with different concentrations of ZnO NPs in 60mmol·L-1NaCl solution showed an increasing trend. Within the germination Dt = 44.25–48 h, the germination rates of radish seeds soaked in Ionized water (CK), 200mg·L^-1^, 400mg·L^-1^, 600mg·L^-1^, and 800mg·L^-1^ZnO NPs were 78.33%, 89.12%, 82.50%, 77.50% and 78.13%, respectively. It can be seen that with the increase of ZnO NPs concentration, the germination rate of radish seeds showed an upward and then downward trend, where the germination rate was the highest when the concentration was 200mg·L^-1^ZnO NPs.

Figure 8 (c) show the changes in germination index and germination potential of radish seeds treated with different concentrations of ZnO NPs in 60mmol·L^-1^NaCl solution with the advancement of time. The germination potential of seeds soaked in CK, 200mg·L^-1^, 400mg·L^-1^, 600mg·L^-1^, 800mg·L^-1^ZnO NPS was 48.63%, 54.17%, 62.33%, 56.17% and 50.00%, respectively. Within the germination Dt = 44.25–48 h, the germination index of the seeds treated with each concentration was 26.45. 33.00, 39.40, 26.93 and 25.52, respectively. It can be seen that with the increase of ZnO NPs concentration, both the germination potential and germination index show an upward and then downward trend. Where, the germination potential and germination index of the seeds were the highest when they were soaked in 400mg·L^-1^ZnO NPs; the germination potential and germination index of the seeds soaked in 600mg·L^-1^ZnO NPs started to decline; and the inhibition effect was more significant when they were socked in 800mg·L^-1^ZnO NPs. However, the germination potential of seeds treated with all concentrations of ZnO NPs was higher that that in CK. Except the seeds treated with 800mg·L^-1^ZnO NPs, the germination index of all seeds treated with rest concentrations of ZnO NPs was higher than that of CK. Therefore, the treatment of radish seeds with low-concentration ZnO NPs in 60mmol·L^-1^NaCl solution can improve the speed and potential of germination.

(4) The effects of different concentrations of ZnO NPs on the germination of radish seeds in 90mmol·L^-1^NaCl solution. The germination test of radish seeds treated with T3, AT3, BT3, CT3 and DT3 were carried out, as shown in Table 4.

Figure 7 (d) shows the change in germination rate of radish seeds with different concentrations of ZnO NPs soaked in 90mmol·L^-1^NaCl solution with the advancement time (the same calculation method in (1) is adopted). As can be seen from Fig. 7 (d), with the advancement of germination Dt, the germination rate of radish seeds treated with different concentrations of ZnO NPs in 90mmol·L-1NaCl solution showed an increasing trend. Within the germination Dt = 44.25–48 h, the germination rates of radish seeds soaked in deionized water (CK), 200mg·L^-1^, 400mg·L^-1^, 600mg·L^-1^, and 800mg·L^-1^ZnO NPs were 48.33%, 63.33%, 57.83%, 55.88% and 54.38%, respectively. It can be seen that with the increase of ZnO NPs concentration, the germination rate of radish seeds showed an upward and then downward trend. Where, the germination rate was the highest when the concentration was 200mg·L^-1^ZnO NPs, and the germination rate of seeds soaked in each concentration of ZnO NPs was higher than that of the control group CK.

Figure 8 (d) show the changes in germination index and germination potential of radish seeds treated with different concentrations of ZnO NPs in 90mmol·L^-1^NaCl solution with the advancement of time. The germination potential of seeds soaked in CK, 200mg·L^-1^, 400mg·L^-1^, 600mg·L^-1^, 800mg·L^-1^ZnO NPS was 41.68%. 46.25%, 48.33%, 39.17% and 35.00%, respectively. Within the germination Dt = 44.25–48 h, the germination index of the seeds treated with each concentration was 17.14, 19.62, 18.44, 17.75 and 14.86, respectively. It can be seen that with the increase of ZnO NPs concentration, both the germination potential and germination index show an upward and then downward trend. Where, the germination potential and germination index of the seeds showed an increasing trend when they were soaked in 200mg·L^-1^ and 400mg·L^-1^ZnO NPs, and reached the maximum value when they were soaked in 400mg·L^-1^ZnO NPs. The germination potential and germination index of the seeds started to decline when they were soaked in 600mg·L^-1^ZnO NPs, and the inhibition effect was more obvious when they were soaked in 800mg·L^-1^ZnO NPs. The germination potential of seeds soaked in 600mg·L^-1^ and 800mg·L^-1^ZnO NPs was lower than that in CK, and the germination index of seeds soaked in 800mg·L^-1^ZnO NPs was lower than that in CK. Therefore, the treatment of radish seeds with low-concentration ZnO NPs in 90mmol·L^-1^NaCl solution can improve the speed and potential of germination, while high-concentration ZnO NPs will inhibit the speed and potential of germination.

(5) The effects of different concentrations of ZnO NPs on the germination of radish seeds in 120mmol·L^-1^NaCl solution. The germination test of radish seeds treated with T4, AT4, BT4, CT4 and DT4 were carried out, as shown in Table 4.

Figure 7(e) shows the change in germination rate of radish seeds with different concentrations of ZnO NPs soaked in 120mmol·L^-1^NaCl solution with the advancement time (the same calculation method in (1) is adopted). As can be seen from Fig. 7 (e), with the advancement of germination Dt, the germination rate of radish seeds treated with different concentrations of ZnO NPs in 120mmol·L-1NaCl solution showed an increasing trend. Within the germination Dt = 44.25–48 h, the germination rates of radish seeds soaked in Ionized water (CK), 200mg·L^-1^, 400mg·L^-1^, 600mg·L^-1^, and 800mg·L^-1^ZnO NPs were 35.13%, 45.00%, 57.50%, 51.38% and 39.69%, respectively. It can be seen that with the increase of ZnO NPs concentration, the germination rate of radish seeds showed an upward and then downward trend, where the germination rate was the highest when the concentration was 400mg·L^-1^ZnO NPs and the germination rate of seeds soaked with different concentrations of ZnO NPs was higher than that in CK.

Figure 8(e) show the changes in germination index and germination potential of radish seeds treated with different concentrations of ZnO NPs in 120mmol·L^-1^NaCl solution with the advancement of time. The germination potential of seeds soaked in CK, 200mg·L^-1^, 400mg·L^-1^, 600mg·L^-1^, 800mg·L^-1^ZnO NPS was 19.58%, 22.50%, 26.17%, 17.92% and 21.25%, respectively. Within the germination Dt = 44.25–48 h, the germination index of the seeds treated with each concentration was 10.12, 11.63, 14.73, 11.86 and 11.25, respectively. It can be seen that with the increase of ZnO NPs concentration, both the germination potential and germination index show an upward and then downward trend. Where, the germination potential and germination index of the seeds were the highest when they were soaked in 200mg·L^-1^ZnO NPs; the germination potential and germination index of the seeds soaked in 600mg·L^-1^ZnO NPs started to decline; and the inhibition effect was more significant when they were socked in 800mg·L^-1^ZnO NPs. The germination potential and germination index of seeds treated with all concentrations of ZnO NPs was higher than those in CK. Therefore, the treatment of radish seeds with low-concentration ZnO NPs in 120mmol·L^-1^NaCl solution can improve the speed and potential of germination.

Finally, according to the longitudinal comparison of the above five sets of data, after the soaking in deionized water, the germination rate of radish seeds cultivated with 0, 30mmol·L^-1^, 60mmol·L^-1^, 90mmol·L^-1^, and 120mmol·L^-1^NaCl solution was 97.50%, 90.00%, 78.33%, 48.33% and 35.13%, respectively. It can be seen that with the increase of salt concentration, its inhibition effect on the germination of radish seeds is more obvious. After the soaking in other concentrations of ZnO NPs, as the concentration of NaCl solution increases, the inhibition effect on germination of radish seeds is more obvious.

## Conclusions

In order to address low efficiency, large error, and proneness to damage to seed structure in the traditional manual testing of seed germination and cater to the needs of non-destructive monitoring of the whole-process germination of radish seeds, the following work has been carried out:

(1) A full time-series sequence seed germination phenotypic collection system consisting of seed cultivation module, environmental control module, image data acquisition module, human-computer interaction module, and phenotypic data analysis module was used. The images of the germination of radish seeds in 16 cells were collected at an interval of 15 min, thereby constructing a label data set of the images of the whole-process germination of radish seeds; the germination tests of radish seeds were conducted on the dual variables of ZnO NPs treatment and salt stress, and the whole-process germination images of radish seeds with 48 h were collected;

(2) Based on the YOLOv8n model, tests were carried out to evaluate the detection precision of the germination images of radish seeds and the model complexity. With replacement of backbone network with PP-LCNet, the adoption of cross-scale feature fusion module (CCFM) in the neck part, the replacement of C2f of the neck part with OREPA, the replacement of SPPF with FocalModulation, and the replacement of Detect with LADH, the YOLOv8n detection model was improved to P-YOLOv8, PC-YOLOv8, PCO-YOLOv8, PCOF-YOLOv8 and YOLOv8-R detection models, and ablation tests were carried out. Then, the detection precision and model complexity of the foregoing models were analyzed; Comparative tests were made among the YOLOv8-R, YOLOv3-tiny, YOLOv5s, YOLOv6-v3.0, YOLOv7-tiny, YOLOv8s and other widely used target detection models, which verified the effectiveness of the above improvement methods;

(3) The germination rate, germination index and germination potential were used as the three indicators for evaluating the vitality. On the basis of the YOLOv8-R model, the germination of radish seeds cultivated in 0, 30mmol·L^-1^, 60mmol·L^-1^, 90mmol·L^-1^, 120mmol·L^-1^NaCl after treatment with CK, 200mg·L^-1,^ 400mg·L^-1^, 600mg·L^-1^, and 800mg·L^-1^ZnO NPs was analyzed. The results show that: after the soaking in deionized water, with the increase of the concentration of NaCl solution, the number of germinated radish seeds gradually decreased, and the germination rate, germination potential and germination index all showed a declining trend; in the cultivation in deionized water, compared with the deionized water (CK) group, with the increase of the concentration of ZnO NPs, the germination rate does not change significantly, and the germination index and germination potential both show an upward and then downward trend; in the cultivation in 30mmol·L^-1^、60mmol·L^-1^、90mmol·L^-1^、120mmol·L^-1^NaCl solutions, compared with the deionized water (CK) group, with the increase of the concentration of ZnO NPs, the germination rate, germination potential and germination index of radish seeds all show an upward and then downward trend. Therefore, the treatment with low-concentration ZnO NPs can promote the germination rate and germination potential of radish seeds under salt stress, and while high-concentration ZnO NPs inhibits their germination rate and germination potential.

It has been verified that the YOLOv8-R model can not only effectively detect radish seeds, but also perform well in identifying pea seeds, proving that YOLOv8-R has the characteristics of cross-domain adaptability. The seed germination vitality detection method based on this model also has high use value. In summary, it is expected that the method for analyzing the whole-process germination of seeds based on the target lightweight YOLOv8-R test model will provide a valuable reference for a deeper understanding of the germination process of soaked crop seeds, the exploration of the influence of nanomaterials on crops’ germination characteristics and internal physiological characteristics, the application of nanomaterials in agriculture and biotechnology, and the breeding of salt-resistant crops, thereby achieving digital modern agriculture and high yield of high-quality crops.

## Data Availability

No datasets were generated or analysed during the current study.

## Abbreviations

ZnO NPs: Zinc Oxide Nanoparticles
YOLO: You Only Look Once
LED: Light Emitting Diode
PLC: Programmable Automation Controller
Adam: Adaptive Moment Estimation
P: Precision
R: Recall
AP: Average Precision
mAP: mean Average precision
FLOPs: Floating-point Operations Per second
IoU: Intersection over Union
NMS: Non-Maximum Suppression

## References

[1] Zhang T, Fan S, Xiang Y, Zhang S, Wang J, Sun Q. Non-destructive analysis of germination percentage, germination energy and simple vigour index on wheat seeds during storage by Vis/NIR and SWIR hyperspectral imaging. Spectrochim Acta A. 2020;239:118488. 10.1016/j.saa.2020.118488. 10.1016/j.saa.2020.118488 32470809

[2] Abebe G, Alemu A. Role of improved seeds towards improving livelihood and food security at Ethiopia. Int J Research-Granthaalayah. 2017;5(2):338–56. 10.29121/granthaalayah.v5.i2.2017.1746. 10.29121/granthaalayah.v5.i2.2017.1746

[3] Feng L, Zhu S, Liu F, He Y, Bao Y, Zhang C. Hyperspectral imaging for seed quality and safety inspection: a review. Plant Methods. 2019;15:91. 10.1186/s13007-019-0476-y. 31406499 10.1186/s13007-019-0476-y PMC6686453

[4] Xia Y, Xu Y, Li J, Zhang C, Fan S. Recent advances in emerging techniques for non-destructive detection of seed viability: a review. Artif Intell Agric. 2019;1:35–47. 10.1016/j.aiia.2019.05.001. 10.1016/j.aiia.2019.05.001

[5] Li X, Wang J, Qiu Y, Wang H, Wang P, Zhang X, Li C, Song G, Gui W, Shen D, Yang W, Cai B, Liu L, Li X. SSR-sequencing reveals the inter-and intraspecific genetic variation and phylogenetic relationships among an extensive collection of Radish (Raphanus) germplasm resources. Biology. 2021;10(12):1250. 10.3390/biology10121250. 34943165 10.3390/biology10121250 PMC8698774

[6] Curtis IS. Genetic engineering of radish: current achievements and future goals. Plant Cell Rep. 2011;30(5):733–44. 10.1007/s00299-010-0978-6. 21191596 10.1007/s00299-010-0978-6

[7] Chieb M, Gachomo EW. The role of plant growth promoting rhizobacteria in plant drought stress responses. Bmc Plant Biol. 2023;23(1):407. 10.1186/s12870-023-04403-8. 37626328 10.1186/s12870-023-04403-8 PMC10464363

[8] Kalpana VN, Devi Rajeswari V. A review on green synthesis, biomedical applications, and toxicity studies of ZnO NPs. Bioinorg Chem Appl. 2018;2018:3569758. 10.1155/2018/3569758. 30154832 10.1155/2018/3569758 PMC6093006

[9] Wang N, Liu D, Xie MW, Li QB, Liu QM. Behavior and toxicity of zinc oxide nanoparticles in aquatic environment. Environ Chem. 2016;35:2528–34.

[10] Kang M, Liu Y, Weng Y, Wang H, Bai X. A critical review on the toxicity regulation and ecological risks of zinc oxide nanoparticles to plants. Environ Sci-Nano. 2024;11:14–35. 10.1039/D3EN00630A. 10.1039/D3EN00630A

[11] Yang Z, Chen J, Dou R, Gao X, Mao C, Wang L. Assessment of the phytotoxicity of metal oxide nanoparticles on two crop plants, maize (Zea mays L.) and rice (Oryza sativa L). Int J Env Res Pub He. 2015;12(12):15100–9. 10.3390/ijerph121214963. 10.3390/ijerph121214963 PMC4690899 26633437

[12] Li Y, Liang L, Li W, Ashraf U, Ma L, Tang X, Pan S, Tian H, Mo Z. ZnO nanoparticle-based seed priming modulates early growth and enhances physio-biochemical and metabolic profiles of fragrant rice against cadmium toxicity. J Nanobiotechnol. 2021;19:75. 10.1186/s12951-021-00820-9. 10.1186/s12951-021-00820-9 PMC7968244 33731120

[13] He X, Feng X, Sun D, Liu F, Bao Y, He Y. Rapid and nondestructive measurement of rice seed vitality of different years using near-infrared hyperspectral imaging. Molecules. 2019;24(12):2227. 10.3390/molecules24122227. 31207950 10.3390/molecules24122227 PMC6630334

[14] Hampton JG, Martinelli AH, Farrant JM, Schmiermann HMJ, Powell AA, Abdelmonem AM, Mtindi K, Come D, Ednie AB. Seed technology-past, present and future. Seed Sci Technol. 1999;27(2):681–98.

[15] Aosa I. Seed vigor testing handbook. Association Official Seed Anal Contribution. 1983;32:88.

[16] Guzmán-Ortiz FA, Castro-Rosas J, Gómez-Aldapa CA, Mora-Escobedo R, Rojas-León A, Rodríguez-Marín ML, Falfán-Cortés RN. Román-Gutiérrez AD. Enzyme activity during germination of different cereals: a review. Food Rev Int. 2019;35(3):177–200. 10.1080/87559129.2018.1514623. 10.1080/87559129.2018.1514623

[17] Borji M, Ghorbanli M, Sarlak M. Some seed traits and their relationships to seed germination, emergence rate, electrical conductivity in common bean (Phaseolus vulgaris L). Asian J Plant Sci. 2007;6(5):781–7. 10.3923/ajps.2007.781.787

[18] Wang Q, Yang M, Pei J, Wang L, Wu YY, Lv H. Effect of moisture content on vigor of Cyathula officinalis seeds and its anti-aging mechanism analysis. Zhongguo Zhong Yao Za Zhi = Zhongguo zhongyao zazhi = China. J Chin Materia Med. 2016;41(7):1222–6. 10.4268/cjcmm20160711. 10.4268/cjcmm20160711 28879735

[19] Kranner I, Kastberger G, Hartbauer M, Pritchard HW. Noninvasive diagnosis of seed viability using infrared thermography. Proceedings of the National Academy of Sciences. 2010;107(8):3912–3917. 10.1073/pnas.09141971. 10.1073/pnas.0914197107 PMC2840516 20133712

[20] Zhang T, Sun Q, Yang L, Yang L, Wang J. Vigor detection of sweet corn seeds by optimal sensor array based on electronic nose. Trans Chin Soc Agricultural Eng. 2017;33(21):275–81.

[21] Braga RA, Dal Fabbro IM, Borem FM, Rabelo G, Arizaga R, Rabal HJ, Trivi M. Assessment of seed viability by laser speckle techniques. Biosyst Eng. 2003;86(3):287–94. 10.1016/j.biosystemseng.2003.08.005. 10.1016/j.biosystemseng.2003.08.005

[22] Genze N, Bharti R, Grieb M, Schultheiss SJ, Grimm DG. Accurate machine learning-based germination detection, prediction and quality assessment of three grain crops. Plant Methods. 2020;16:1–11. 10.1186/s13007-020-00699-x. 33353559 10.1186/s13007-020-00699-x PMC7754596

[23] Nehoshtan Y, Carmon E, Yaniv O, Ayal S, Rotem O. Robust seed germination prediction using deep learning and RGB image data. Sci Rep. 2021;11(1):22030. 10.1038/s41598-021-01712-6. 34764422 10.1038/s41598-021-01712-6 PMC8586350

[24] Toda Y, Okura F, Ito J, Okada S, Kinoshita T, Tsuji H, Saisho D. Training instance segmentation neural network with synthetic datasets for crop seed phenotyping. Commun Biol. 2020;3(1):173. 10.1038/s42003-020-0905-5. 32296118 10.1038/s42003-020-0905-5 PMC7160130

[25] Zhao J, Ma Y, Yong K, Zhu M, Wang Y, Luo Z, Wei X, Huang X. (2023). Deep-learning‐based automatic evaluation of rice seed germination rate. J Sci Food Agr. 2023;103(4):1912–1924. 10.1002/jsfa.12318. 10.1002/jsfa.12318 36335532

[26] Zhang M, Zhao J, Hoshino Y. Deep learning-based high-throughput detection of in vitro germination to assess pollen viability from microscopic images. J Exp Bot. 2023;74(21):6551–62. 10.1093/jxb/erad315. 37584205 10.1093/jxb/erad315 PMC10662222

[27] Bai WW, Zhao XN, Luo B, Zhao W, Huang S, Zhang H. Research on wheat seed germination detection method based on Yolov5. Acta Agricult Zhejiangensis. 2023;35:445–54.

[28] Jiang H, Hu F, Fu X, Chen C, Wang C, Tian L, Shi Y. Yolov8-Peas: a lightweight drought tolerance method for peas based on seed germination vigor. Front Plant Sci. 2023;14:1257947. 10.3389/fpls.2023.1257947. 37841608 10.3389/fpls.2023.1257947 PMC10568755

[29] Terven J, Cordova-Esparza D. A comprehensive review of YOLO: from YOLOv1 and beyond. arXiv. 2023;2023. arXiv:2304.00501. arXiv preprint.

[30] Cui C, Gao T, Wei S, Du Y, Guo R, Dong S, Lu B, Zhou Y, Lv X, Liu Q, Hu X, Yu D, Ma Y. PP-LCNet: a lightweight CPU convolutional neural network. ArXiv Preprint ArXiv: 2109. 15099, 2021. 10.48550/arXiv.2109.15099.

[31] Howard AG, Zhu M, Chen B, Kalenichenko D, Wang W, Weyand T, Andreetto M, Adam H. Mobilenets: efficient convolutional neural networks for mobile vision applications. ArXiv Preprint ArXiv: 1704. 04861, 2017. 10.48550/arXiv.1704.04861.

[32] Howard A, Sandler M, Chu G, Chen LC, Chen B, Tan M, Wang W, Zhu Y, Pang R, Vasudevan V, Le QV, Adam H. Searching for mobilenetv3. Proceedings of the IEEE/CVF international conference on computer vision. 2019:1314–1324.

[33] Hu J, Shen L, Sun G. Squeeze-and-excitation networks. in: Proceedings of the IEEE conference on computer vision and pattern recognition. 2018:7132–7141.

[34] Zhao Y, Lv W, Xu S, Wei J, Wang G, Dang Q, Liu Y, Chen J. Detrs beat yolos on real-time object detection. ArXiv Preprint ArXiv: 2304. 2023;08069. 10.48550/arXiv.2304.08069.

[35] Hu M, Feng J, Hua J, Lai B, Huang J, Gong X, Hua XS. Online convolutional re-parameterization. Proceedings of the IEEE/CVF conference on computer vision and pattern recognition. 2022:568–577.

[36] Yang J, Li C, Dai X, Gao J. Focal modulation networks. Adv Neural Inf Process Syst. 2022;35:4203–17.

[37] Yang J, Li C, Zhang P, Dai X, Xiao B, Yuan L, Gao J. Focal attention for long-range interactions in vision transformers. Adv Neural Inf Process Syst. 2021;34:30008–22.

[38] Liang J, Jiang L, Cao L, Kalantidis Y, Li LJ, Hauptmann AG. Focal visual-text attention for memex question answering. IEEE Trans Pattern Anal Mach Intell. 2019;41(8):1893–908. 10.1109/TPAMI.2018.2890628. 30624212 10.1109/TPAMI.2018.2890628

[39] Zhang S, Chi C, Yao Y, Lei Z, Li SZ. Bridging the gap between anchor-based and anchor-free detection via adaptive training sample selection. Proceedings of the IEEE/CVF conference on computer vision and pattern recognition. 2020: 9759–9768.

[40] Bodla N, Singh B, Chellappa R, Davis LS. Soft-NMS–improving object detection with one line of code. Proceedings of the IEEE international conference on computer vision. 2017:5561–5569.

[41] Wang G, Chen Y, An P, Hong H, Hu J, Huang T. UAV-YOLOv8: a small-object-detection model based on improved YOLOv8 for UAV aerial photography scenarios. Sensors. 2023;23(16):7190. 10.3390/s23167190. 37631727 10.3390/s23167190 PMC10458807

[42] Jouyban Z. The effects of salt stress on plant growth. Tech J Eng Appl Sci. 2012;2(1):7–10.

[43] Siddiqui MH, Al-Whaibi MH, Firoz M, Al-Khaishany MY. Role of nanoparticles in plants. Nanatechnol Plant Sciences: Nanopart Their Impact Plants. 2015;19–35. 10.1007/978-3-319-14502-0_2.

